# Efficacy of imiquimod 5% cream as first-line management in cutaneous leishmaniasis caused by *Leishmania mexicana*

**DOI:** 10.1590/0037-8682-0305-2020

**Published:** 2021-03-22

**Authors:** Gabriela Fuentes-Nava, Andrés Tirado-Sánchez, Edith A. Fernández-Figueroa, Sokani Sánchez-Montes, Ingeborg Becker, Alexandro Bonifaz

**Affiliations:** 1 Hospital General de México, Servicio de Dermatología, México.; 2 Instituto Mexicano del Seguro Social, Hospital General de Zona 29, Departamento de Medicina Interna, México.; 3 Instituto Nacional de Medicina Genómica, Laboratorio de Genómica Computacional, México.; 4 Universidad Nacional Autónoma de México, Facultad de Medicina, Centro de Medicina Tropical, Unidad de Medicina Experimental, México.; 5 Universidad Veracruzana, Facultad de Ciencias Biológicas y Agropecuarias, México.

**Keywords:** Leishmaniasis, Imiquimod, Treatment

## Abstract

Cutaneous leishmaniasis (CL) involves several differential diagnoses as it lacks a gold standard diagnostic test. Its diagnosis is easier in endemic regions; however, many cases come from travelers to endemic areas. A 22-year-old patient, who had recently visited Oaxaca, Mexico, developed two asymptomatic ulcers weeks later on the left auricle and the nose. *Leishmania mexicana* was identified using polymerase chain reaction. The patient was treated with imiquimod 5% cream three times/week, providing favorable results after 12 weeks, without relapse 2 months after therapy. To our knowledge, this is the first case of CL due to *L. mexicana* effectively treated with imiquimod.

## INTRODUCTION

Leishmaniasis is an infectious disease caused by different intracellular protozoal species related to the genus *Leishmania (L.)* and transmitted to humans from a female hematophagous of the Diptera order from the genus *Lutzomyia* (in America) or *Phlebotomus* (in Europe, Asia, and Africa)^1^ ^,^ ^2^, which regurgitates promastigotes. Leishmaniasis can be classified into three primary clinical forms: cutaneous (localized or diffused), mucocutaneous, and visceral leishmaniasis (or kala-azar)^1^ ^,^ ^3^. In Mexico, cutaneous leishmaniasis is endemic to Campeche, Chiapas, Coahuila, Jalisco, Michoacán, Nayarit, Nuevo León, Oaxaca, Tamaulipas, Quintana Roo, Tabasco, Veracruz, Yucatán, and Sinaloa^4^. Several treatments have been tested for cutaneous leishmaniasis with different outcomes. Imidazoquinolines, including imiquimod, are low-molecular-weight substances that can regulate immune responses through interactions with toll-like receptors on cells. Imiquimod acts via immunomodulatory activity on several immune cells (Langerhans cells), regulating the release of proinflammatory cytokines (IFN-γ, TNF-α, IL-1β, IL-1α, IL-6, IL-1) receptor antagonists, granulocyte-macrophage colony-stimulating factor and granulocyte colony-stimulating factor and also regulates nitric oxide-mediated phagocytosis^5^. Although other topical to intralesional alternatives for the management of cutaneous leishmaniasis, such as pentamidine isethionate or pentavalent antimonial have been tested with good results, many of these are not available in Mexico; therefore, the search for new treatment options is required. We report the first case of localized cutaneous leishmaniasis caused by *L. mexicana* successfully treated with imiquimod 5% cream as the first-line therapy. 

## CASE REPORT

**FIGURE 1:**
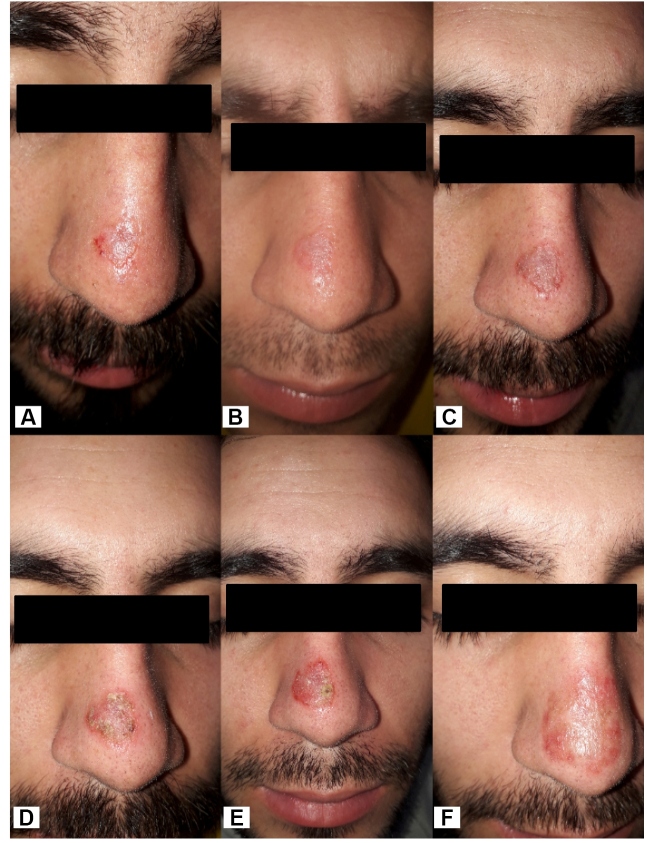
**(A)** a 13 × 18 mm ulcerated nodule in the dorsum of the nose; **(B)** Two weeks of treatment with imiquimod 5% cream; **(C)** Four weeks of treatment; **(D)** Eight weeks of treatment; **(E)** Twelve weeks of treatment; **(F)** Two months after termination of treatment.

**FIGURE 2:**
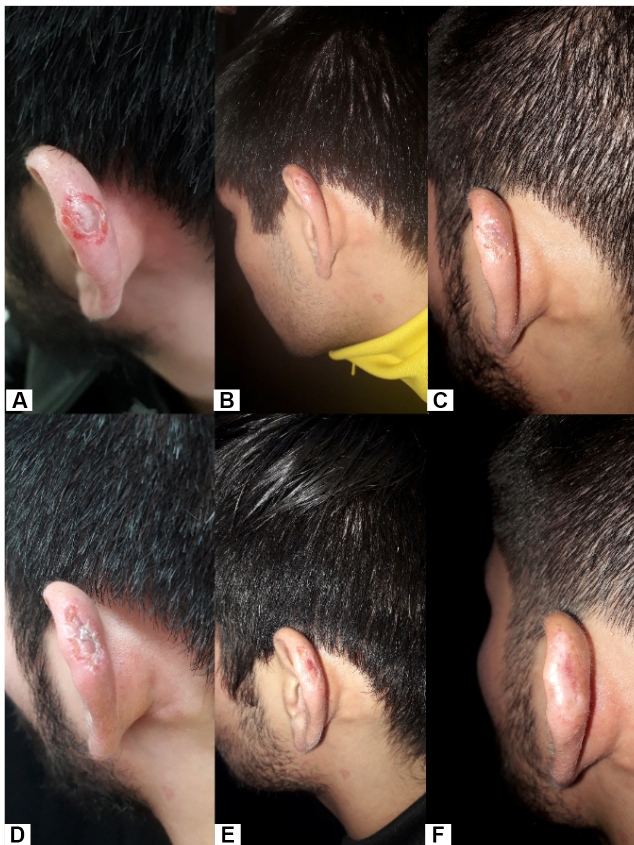
**(A)** an ulcerated nodule in the left atrial pavilion; **(B)** Two weeks of treatment with imiquimod 5% cream; **(C)** Four weeks of treatment; **(D)** Eight weeks of treatment; **(E)** Twelve weeks of treatment; **(F)** Two months after termination of treatment.

**FIGURE 3:**
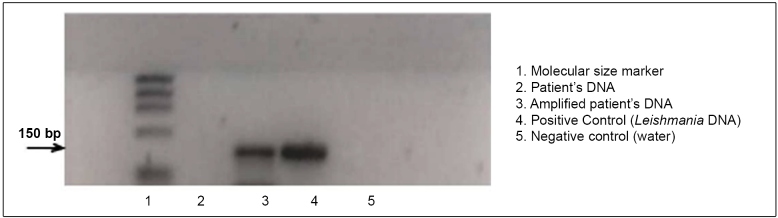
DNA extraction was done using the High Pure PCR Template Preparation Kit (Roche, Cat.11796828001). The DNA integrity of the clinical sample was evaluated with human glyceraldehyde-3-phosphate dehydrogenase (GAPDH) and it was amplified by qPCR. As positive controls, DNA isolated from *Leishmania amazonensis* MHOM/BR/00/LTB0016, *L*. *amazonensis* IFLA/BR/67/PH8, *L. mexicana* MHOM/MX/2011/Lacandona, and *L. mexicana* isolate 14 were used as templates. The sample was positive for *L. mexicana.* Labels: 1.Molecular size marker; 2.Patient’s DNA; 3.Amplified patient´s DNA; 4.Positive control (*Leishmania* DNA); and, 5.Negative control (water).

A previously healthy 22-year-old man presented to our dermatology department with an 8-week skin lesion that developed 20 weeks after traveling to the Chacahua Lagoons in the state of Oaxaca. We observed a 13 × 18 mm ulcerated nodule in the dorsum of the nose and left ear (Figure 1a and Figure 2a, respectively). The patient was previously treated with antibiotics without success. An ear biopsy (6 mm) was performed followed by DNA extraction using the High Pure PCR Template Preparation Kit (Roche, Cat.11796828001). Human glyceraldehyde-3-phosphate dehydrogenase was amplified by quantitative PCR (qPCR), as reported previously^6^, to evaluate the DNA integrity of the clinical sample. Further, a qPCR assay (qPCR-ama) targeting kDNA minicircles was performed as previously described^7^. As positive controls, DNA isolated from *L*. *amazonensis* MHOM/BR/00/LTB0016, *L*. *amazonensis* IFLA/BR/67/PH8, *L. mexicana* MHOM/MX/2011/Lacandona, and *L. mexicana* isolate 14 were used as templates. Non-template reactions were used as negative controls. The sample tested positive for *L. mexicana* (Figure 3). The treatment options were formulated based on the size of the lesion; however, the patient refused systemic treatment; therefore, we decided to prescribe 5% imiquimod cream, achieving a favorable response at 12 weeks (Figure 1b-e; Figure 2b-e) and no relapse at the 2-month follow-up (Figure 1f, Figure 2f).

## DISCUSSION

Leishmaniasis is a parasitic infection with an uncertain frequency. Localized cutaneous leishmaniasis initially develops as a pruritic papule, progressing to an ulcer in exposed areas such as the ears, face, or arms. The definitive diagnosis is the identification of amastigotes inside the phagocytic cells isolated from the lesion by a stained imprint that facilitates microscopic identification or molecular tests (PCR).

Our results are consistent with those of previous reports. Córdoba-Uscanga et al.^4^, showed that *L. mexicana* is the most common causative agent of cutaneous leishmaniasis in Mexico. 

Topical imiquimod, an immunomodulator and antineoplastic agent, has been used in the management of cutaneous leishmaniasis in two main contexts, that is, in combination with other treatments such as antimonials and cases resistant to conventional treatments, due to the leishmanicidal activity demonstrated by it in tissue culture and experimental studies^8^. 

The management of cutaneous leishmaniasis is a therapeutic challenge due to the toxicity of the drugs and drug resistance; therefore, the treatment must be individualized according to disease severity, the etiological agent, and the host.

In cutaneous leishmaniasis, imiquimod stimulates interferon-γ secretion by CD4 T helper-1 lymphocytes by activating macrophages to destroy amastigotes^9^. This mechanism of action could reduce resistance and treat areas with surrounding subclinical infection. In our patient, a moderate inflammatory reaction, which extended beyond the initial lesion, was observed at 4 weeks of use and was solved after 6 weeks without additional treatment or termination of the imiquimod treatment.

Some *in vivo* and *in vitro* ^10^reports have shown the effect of topically applied imiquimod on cutaneous leishmaniasis^10^ ^-^ ^12^. However, a recent systematic review established that imiquimod has no significant therapeutic effect when combined with antimonials^13^. Our patient achieved complete recovery after topical imiquimod treatment without previous treatments, and no relapse was observed after a 2-month follow-up. Moreover, spontaneous healing of cutaneous leishmaniasis may also occur, and this factor cannot be excluded in the present case; however, the treatment reduced the growth of the lesions, limiting complications, mainly scarring, and sped up the healing process. 

To our knowledge, this is the first case of cutaneous leishmaniasis due to *L. mexicana* successfully treated with 5% imiquimod as a first-line therapy.
